# ‘A seamless transition’: how to sustain a community health worker scheme within the health system of Gombe state, northeast Nigeria

**DOI:** 10.1093/heapol/czab063

**Published:** 2021-06-15

**Authors:** Deepthi Wickremasinghe, Yashua Alkali Hamza, Nasir Umar, Barbara Willey, Magdalene Okolo, Ahmed Gana, Abdulrahman Shuaibu, Jennifer Anyanti, Tanya Marchant, Neil Spicer

**Affiliations:** London School of Hygiene and Tropical Medicine, Keppel Street, London, WC1E 7HT, UK; Childcare and Wellness Clinics, 26 Anthony Enahoro Street, Utako, Abuja, Nigeria; London School of Hygiene and Tropical Medicine, Keppel Street, London, WC1E 7HT, UK; London School of Hygiene and Tropical Medicine, Keppel Street, London, WC1E 7HT, UK; Formerly with Society for Family Health, Justice Ifeyinwa Nzeako House, #8 Port Harcourt Crescent, Area 11, Garki, Abuja, Nigeria; Gombe State Government, Dukku Road, Gombe, Nigeria; Gombe State Primary Health Care Development Agency, Jeka Da Fari, near Government science secondary school, 760212 Gombe, Nigeria; Society for Family Health, Justice Ifeyinwa Nzeako House, #8 Port Harcourt Crescent, Area 11, Garki, Abuja, Nigeria; London School of Hygiene and Tropical Medicine, Keppel Street, London, WC1E 7HT, UK; London School of Hygiene and Tropical Medicine, Keppel Street, London, WC1E 7HT, UK

**Keywords:** Sustainability, community health workers, health systems, Nigeria

## Abstract

Health interventions introduced as part of donor-funded projects need careful planning if they are to survive when donor funding ends. In northeast Nigeria, the Gombe State Primary Health Care Development Agency and implementing partners recognized this when introducing a Village Health Worker (VHW) Scheme in 2016. VHWs are a new cadre of community health worker, providing maternal, newborn and child health-related messages, basic healthcare and making referrals to health facilities. This paper presents a qualitative study focussing on the VHW Scheme’s sustainability and, hence, contributes to the body of literature on sustaining donor-funded interventions as well as presenting lessons aimed at decision-makers seeking to introduce similar schemes in other Nigerian states and in other low- and middle-income settings. In 2017 and 2018, we conducted 37 semi-structured interviews and 23 focus group discussions with intervention stakeholders and community members. Based on respondents’ accounts, six key actions emerged as essential in promoting the VHW Scheme’s sustainability: government ownership and transition of responsibilities, adapting the scheme for sustainability, motivating VHWs, institutionalizing the scheme within the health system, managing financial uncertainties and fostering community ownership and acceptance. Our study suggests that for a community health worker intervention to be sustainable, reflection and adaption, government and community ownership and a phased transition of responsibilities are crucial.

**Key messages**

- Donor-funded maternal and newborn health interventions are commonly not sustained after donor funding ends.
- To sustain maternal and newborn health interventions, reflection and adaption, government and community ownership and a phased transition of responsibilities are crucial.

## Tribute to Deepthi Wickremasinghe

This article is based on the work of its lead author, Deepthi Wickremasinghe, who passed away in April 2020. Deepthi specialized in qualitative research, systematic literature reviews, and information and knowledge management. Deepthi’s many academic contributions included her qualitative research on sustaining maternal and newborn child interventions. She had the ability to present research findings precisely and powerfully for different audiences including policymakers, practitioners and academics. Deepthi’s work is very well received, and she made significant contributions to knowledge about maternal and newborn child health. As well as her academic contributions, Deepthi was always very keen to ensure her work was relevant and valuable to policy decision makers and practitioners – and so would have a positive impact on people’s lives. She led a study about a ‘Village Health Worker’ scheme in Gombe state, Nigeria, and this work forms the basis of this article. The scheme increased community health workers’ skills and capacity and was seen as a significant way to save the lives of mothers and babies. An important part of Deepthi’s work was to give stakeholders in Nigeria real-time feedback on emerging findings as the project developed. And so, Deepthi contributed very directly to the scheme’s success. Deepthi made important scientific contributions to our understanding of sustainability for improved health and was a conscientious, kind, gentle and committed colleague who is greatly missed.

## Introduction

The world is not on target to achieve the health-related sustainable development goals (WHO, 2018), which underscores the importance of developing new and innovative ways to improve health that can be effectively implemented in low-resource settings (Jha *et al.*, 2016). This is where donor funding can play a valuable role: supporting the development of health interventions to ‘test’ what works well and persuading country governments to adopt, scale and sustain the best ones. Yet it is common that donor-funded interventions are not adopted, scaled and sustained, or, over time, they diminish in efficacy or deviate from the intended protocol (Chambers *et al.*, 2013; Iwelunmor *et al.*, 2016; Sgaier *et al.*, 2013; Shelton *et al.*, 2018; Stirman *et al.*, 2012). As well as being wasteful of resources and time, this is counterproductive if it erodes community trust in donor activities (Shediac-Rizkallah and Bone, 1998). Hence, an important concern within health systems research is how to sustain donor-funded health interventions (Chambers *et al.*, 2013; Iwelunmor *et al.*, 2016; Scheirer and Dearing, 2011; Shediac-Rizkallah and Bone, 1998; Stirman *et al.*, 2012).

Multiple factors influence the sustainability of donor-funded health interventions in low-resource settings. These include how interventions are designed, such as their effectiveness, costs, suitability for the skills and attitudes of health workers and potential for adaptation to different local contexts (Chambers *et al.*, 2013; Pallas *et al.*, 2013; Scheirer and Dearing, 2011; Shediac-Rizkallah and Bone, 1998; Shelton *et al.*, 2018; Stirman *et al.*, 2012). Actions taken when interventions are introduced are also important, such as planning for sustainability from the outset, building relationships with stakeholder groups and harnessing powerful champions’ support, as well as ensuring interventions are institutionalized within host health systems rather than existing in parallel (Braithwaite *et al.*, 2018; Chambers *et al.*, 2013; Hirschhorn *et al.*, 2013; Iwelunmor *et al.*, 2016; Larson *et al.*, 2014; Sgaier *et al.*, 2013; Shediac-Rizkallah and Bone, 1998; Shelton *et al.*, 2018; Stirman *et al.*, 2012; Torpey *et al.*, 2010; WHO and ExpandNet, 2010). Aspects of contextual environments into which interventions are introduced can influence sustainability. These include organizational settings, such as health systems capacity to support new interventions, and aspects of broader country contexts, such as the availability of financial resources, supportive leadership, policies, legislation and regulatory institutions and good coordination within government and between multiple actors (Hirschhorn *et al.*, 2013; Larson *et al.*, 2014; Scheirer and Dearing, 2011; Schell *et al.*, 2013; Shediac-Rizkallah and Bone, 1998; Shelton *et al.*, 2018; Stirman *et al.*, 2012; Torpey *et al.*, 2010; Wickremasinghe *et al.*, 2018; WHO and ExpandNet, 2010).

Introducing community health workers such as village health workers (VHWs), and strengthening their roles is a common form of intervention funded by donors to tackle maternal, newborn and child health issues, and there is a growing literature on their effectiveness and sustainability (for example, Black *et al.*, 2017; Pallas *et al.*, 2013; Perry and Crigler *et al.*, 2014). Following the Ouagadougou Declaration on Primary Health Care and Health Systems in Africa (WHO Regional Office for Africa, 2008), the National Primary Health Care Development Agency (NPHCDA) in Nigeria drew up policies under the vision of ‘Primary Health Care Under One Roof’. It included a national roadmap for introducing a new cadre of community health workers, to be known as VHWs in Nigeria’s states, to better link households and the health system (Government of Nigeria, 2014). Based on NPHCDA guidance, the Gombe State Primary Health Care Development Agency (the Agency) introduced a VHW Scheme in September 2016, funded by the Bill and Melinda Gates Foundation and implemented by a nongovernmental implementer, Society for Family Health.

Our paper explores factors at different levels of the health system contributing to the VHW Scheme’s sustainability. Based on semi-structured interviews and focus group discussions with different stakeholder groups, our aim is to identify and describe the key actions that promoted the scheme’s sustainability. This study contributes to the literature on sustaining donor-funded interventions in low-resource settings and provides lessons aimed at decisions-makers seeking to introduce similar schemes into the health systems of other Nigerian states and beyond.

## Methods

### Intervention

**Figure 1.**
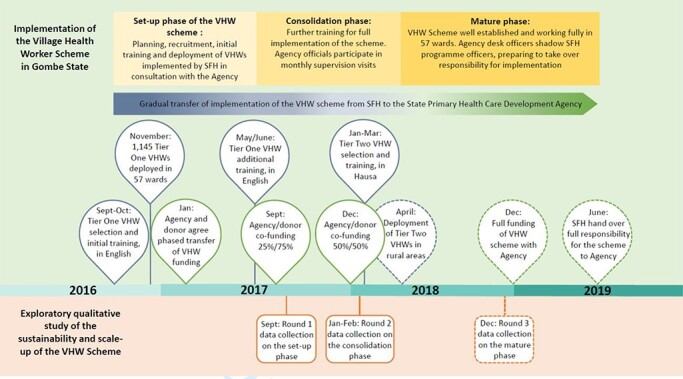
Village Health Worker Scheme’s implementation phases and data collection rounds

Initially the scheme was implemented in 57 of Gombe’s 114 Wards to test and refine it before subsequent scale-up throughout the state. The Agency planned to recruit approximately 1200 VHWs across these wards, serving an estimated population of 1 628 481 (Nigeria, 2006). VHW selection criteria were they were women, aged between 18 and 49 years, preferably married and literate in English. VHWs were expected to work in their own communities, and their role involved delivering maternal, newborn and child healthcare messages; encouraging improved health and healthcare seeking behaviours; undertaking basic healthcare provision, such as treating pregnant women for anaemia and referring women to health facilities, thereby promoting healthcare uptake. They received 4 weeks’ training, a small stipend, a uniform and various job aids. VHWs were directly supervised by Community Health Extension Workers (CHEWs) who provided a link between primary health facilities and the communities they served. CHEWs, a cadre that is specific to Nigeria, are trained for 3 years and deliver basic health services in primary healthcare clinics and in the community. In addition, the work of VHWs was reviewed and discussed by Ward Development Committees (WDCs), a community management structure introduced in Nigeria to oversee the delivery of health and development services, and to represent their communities. An ‘adaptive management process’ was adopted whereby stakeholders periodically reflected on progress and, when necessary, adapted the intervention’s design to operate more effectively. The process involved three phases (Figure 1). In collaboration with the Agency, the ‘set-up phase’ involved the nongovernmental implementer recruiting and training VHWs, and agreeing a phased transition whereby the Agency would take responsibility of implementation and finances. During the ‘consolidation phase’, VHWs received further training, the scheme became fully operational, and the transition began where the Agency’s funding contribution increased incrementally. Finally, the ‘mature phase’ involved the full handover of implementation and financing responsibilities from the nongovernmental implementer to the Agency.

### Study design

We embraced a health policy and systems research approach using qualitative methods. The health policy and systems research approach was appropriate to our aim as the focus is on understanding the influence of policy processes including policy and programme development and implementation, and on intervention outputs rather than measuring outcomes and impacts (Gilson, 2012). We conducted three rounds of in-depth interviews and focus group discussions in 2017 and 2018, which allowed us to trace the scheme’s development over time. Our data collection points aligned with the scheme’s implementation phases (Figure 1). During the ‘set-up phase’, our interviews and focus group discussions explored how sustainability featured in the scheme’s planning and set-up. In the ‘consolidation phase’, we focussed on adaptations aiming to improve the scheme’s sustainability. We conducted a final round of interviews and focus group discussions during the ‘mature phase’, where we asked respondents to reflect on the transition process and the handover of responsibilities to the Agency. After each round, we presented emerging findings to the Agency, the nongovernmental implementer and the donor in the form of oral presentations and research briefs. Hence, the researchers acted as ‘critical friends’, which enhanced our access to research participants and meant our findings could benefit the scheme as it developed (Coghlan and Brydon-Miller, 2014). Nevertheless, this may also have impacted on the data we collected, and our ability to fully capture more negative and critical aspects of the scheme.

### Framework

Our focus was on identifying and better understanding the key actions promoting the scheme’s sustainability. Our interviews and focus group discussions were informed by the literature on sustainability, specifically Hirschhorn *et al.* (2013); Larson *et al.* (2014); Torpey *et al.* (2010); WHO and ExpandNet (2010). Specifically, we explored the following themes:

- **Intervention design:** consideration of sustainability in the scheme’s design;
- **Health worker motivation:** factors motivating VHWs to routinize new practices and remain within the scheme;
- **Institutionalization**: whether and how the scheme was embedded within the health system;
- **Financial and political sustainability:** the availability of adequate resources and political support for the scheme;
- **Social sustainability:** community ownership and acceptance of the scheme.

### Sample

**Figure 2.**
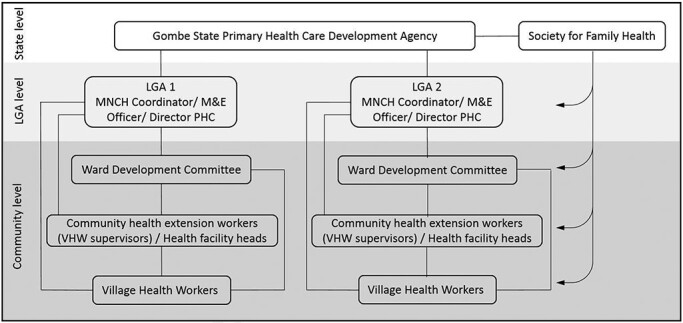
Stakeholder groups at different levels of the health system

Data collection focused on two of Gombe’s 11 Local Government Areas (LGAs), Kaltungo and Nafada, purposively selected as those with the highest and lowest facility deliveries, giving us insights into contrasting health system contexts. Within each LGA, we selected the ward with the best VHW performance, based on monitoring data from the scheme’s first 6 months, because our focus was on identifying the actions promoting the scheme’s sustainability, rather than challenges or failures. Interviews and focus group discussions were conducted with stakeholder groups and beneficiary communities that had experienced the scheme (Figure 2 and Table 1). The focus group discussions, which were moderated by experienced researchers, involved between six and twelve participants, and focussed on intervention design, health worker motivation and social sustainability. Our respondents represented all of the major stakeholder organizations at different levels of the health system involved in the implementation of the scheme. Individuals within those organizations were purposively selected based on their direct involvement and therefore detailed knowledge of the scheme. All VHWs were women. Most CHEWs were women, although two were men. Stakeholders and WDC members were both women and men. In each LGA, we approached community and religious leaders to help us to recruit willing community participants within those areas. Those leaders endorsed our work and encouraged community members to participate but did not influence which community members we invited to be part of our focus group discussions. Refreshments were given to participating VHWs and communities, and community members were each given a bar of soap.

Key: LGA = Local Government Area; MNCH = Maternal, newborn and child health; M&E = Monitoring and evaluation; PHC = Primary healthcare; VHW = Village Health Worker.

**Table 1.** Number of interviews and focus group discussions

Round of interviews	When	Phase	Stakeholder groups interviewed	Number of interviews	Number of FGDs
1	Sept 2017	Setting-up Phase	Donor officer	1	
			Government officials	4	
			Nongovernmental implementers	6	
			Local Government Area officers		2
			Ward Development Committee members		2
			Community Health Extension Workers		2
			Village Health Workers		2
2	Jan–Feb 2018	Consolidation Phase	Government officials	4	
			Nongovernmental implementers	8	
			Ward Development Committee members		2
3	Nov–Dec 2018	Mature Phase	Government officials	6	
			Nongovernmental implementers	8	
			Local Government Area officers		3
			Ward Development Committee members		2
			Community Health Extension Workers		2
			Village Health Workers		2
			Pregnant women		2
			Husbands/partners		2

### Data collection

Informed by our framework, we created a topic guide to explore key themes with our interviewees and focus group discussion respondents, while being attentive to emerging themes. Hence, our approach was both deductive and inductive (Pope and Mays, 2000). We created versions of the topic guide for each stakeholder group, with questions tailored for each round of interviews and focus group discussions. A team of Gombe-based researchers experienced in qualitative interviewing and focus group discussions assisted with the data collection. They were orientated about the study’s purpose, the topic guide and requirements relating to research ethics; they also helped to refine and modify the topic guide. Interviews and focus group discussions were conducted in English or Hausa, based on participants’ preferences. While in the field, emerging themes for follow-up in future interviews and focus group discussions were discussed during daily debriefings. The recordings were transcribed, and where necessary, translated into English.

### Analysis

For each round of data collection, the researchers conducted initial analysis by structuring interview and focus group discussion transcripts according to the major themes within our framework. We deliberately ensured the themes within the framework were broad, and hence, the data collection process was open to emerging themes, rather than confined to rigid categories. Hence, we took a primarily deductive approach while drawing out issues emerging within each theme within our framework. Thematic analysis was then formally conducted by the first author after each round using NVivo 11 to code all of the transcripts. We adopted different approaches to enhance the reliability and validity of our findings (Seale, 2017). We conducted reliability checks of a sample of sound recordings against corresponding transcripts. During daily debriefings, the research team including the first author discussed emerging findings to help triangulate them. The first author also shared and discussed all emerging findings based on NVivo coding with the research team in order to reach a shared interpretation. Reporting emerging findings to stakeholders allowed us to refocus the study after each round of data collection and strengthen the validity of our findings through member checking. In practice, these stakeholders agreed with our findings, and hence, no changes were made on this basis.

### Consent and ethical approval

Before every interview and focus group discussion, each participant was informed about the study and gave written or recorded consent (Hausa or English), including whether we could use sound recorders and include quotations in our outputs. We explained that participants could withdraw from the study at any time. Each day, recordings, field notes, transcripts and consent forms were stored securely on password protected computers. Ethical approval was granted by the authors’ institutes.

## Results

By June 2020, a year after the Agency took full responsibility, a senior representative of the Agency confirmed that the scheme was sustained in the 57 wards of Gombe and a total of 1200 VHWs were fully operational and that they also received additional training and were incorporated within a broader Community Health Influencers Promoters and Services (CHIPS) programme to become ‘CHIPS agents’. Our respondents representing different levels of the health system identified the key actions promoting the scheme’s sustainability: government ownership and transition of responsibilities; adapting the scheme for sustainability; motivating VHWs; institutionalizing the scheme within the health system; managing financial uncertainties and fostering community ownership and acceptance. Based on the interviews and focus group discussions we also reflect on aspects of the capacity of Gombe’s health system that represented challenges to the scheme’s sustainability.

### Government ownership and transition of responsibilities: ‘there’s been a complete transfer of roles…’

The first key action was to ensure that the state government owned the scheme from its inception. Interviewees representing the government, nongovernmental implementer and donor described the scheme as government led, with support from a nongovernmental implementer with donor funding, and hence, the Agency contributed to all major decisions during the scheme’s planning, inception and implementation, and its responsibilities were clarified and decided in advance. Hence, as a government official suggested, ‘seamless transition’ was planned to happen before the nongovernmental implementers’ grant ended in mid-2019, at which time full responsibility for leadership and implementation, with the nongovernmental implementer’s technical support, would pass to the Agency, including full budgetary responsibilities. A senior director supported the process at the Agency; interviewees representing the government and the nongovernmental implementer observed that this strengthened government ownership considerably. There was a phased handover of tasks, involving Agency staff shadowing nongovernmental implementer staff at state and LGA levels. Specifically, government Maternal and Child Health Coordinators and Local Desk Officers worked alongside nongovernmental officers: ‘It falls in perfectly because the [Maternal and Child Health Coordinator] is a senior officer that takes care of maternal and child health in the LGA’, a nongovernmental implementer reflected. By the end of 2018, a nongovernmental interviewee confirmed: ‘…there’s been a complete transfer of roles…to the Agency’.

Government officials and nongovernment implementers highlighted a number of steps that were important in managing the transition process and anticipate potential financial uncertainties. Through a memorandum of understanding between the Agency, the donor and the nongovernmental implementer, the stepped transfer of financial responsibilities to the Agency was agreed. A government official confirmed: ‘We’re actually capturing the scheme in our 2017 budget, which has given us a lot of encouragement…it means government has committed’. The creation of a line item in the state’s health budget was also described by government respondents as vital as it symbolically underlined government commitment to stepping up its financial contributions. Indeed, representatives of the Agency confirmed that it had contributed finances as planned: from 25% to 75% between 2017 and 2018. Yet, notwithstanding this commitment, the Agency is required to request the release of funds from the state Ministry of Finance. Government respondents warned that delays, which were common, could jeopardize the scheme’s sustainability—hence, this reinforced the importance of the steps that were taken to manage the uncertainty: ‘The major weakness is resources…especially financial resources’, a state Government official admitted.

### Adapting the scheme for sustainability: ‘you may end up not getting a single person’

Our respondents representing the government, the nongovernmental implementer and WDCs highlighted a second key action: the ‘adaptive management process’, involving planned, regular reflection points and adapting the scheme’s design where necessary. These respondents confirmed that a critical aspect of this approach was joint decision-making between the Agency, the nongovernmental implementer, and the donor, with inputs from WDCs described below, and that the most important adaptation related to the criteria for recruiting women as VHWs. The intervention required the deployment of 1200 VHWs across half of Gombe’s wards. However, initially, particularly in more remote wards, the English literacy requirement made it impossible to recruit sufficient VHWs. Despite initial enthusiasm that the English literacy requirement could encourage female education, respondents representing the nongovernmental implementer and beneficiary communities suggested that this limited the scheme’s reach. For example, a WDC member observed: ‘…if it proceeds this way…you may end up not getting a single person’. Hence, the selection criteria were amended in 2018 to require literacy in Hausa, the most prevalent language in Gombe and part of the school curriculum, but not necessarily in English. Respondents from all stakeholder groups broadly supported this change: ‘[VHWs] do all things in Hausa. They even talk to [women] in their own local dialects’, explained one LGA officer. Additionally, the preference for married women became a requirement, and the age range was adjusted from 15 to 49 to 18 to 49 years. These ‘tier two’ VHW recruits were trained for exactly the same role. Requiring slightly older, married women helped to address early problems of attrition among VHWs who married and moved to new communities or enrolled in school. WDC members and VHWs themselves confirmed that these new criteria also promoted community acceptance of VHWs, who were recruited closer to their own communities, and married women would be more respected.

### Motivating VHWs: ‘we enjoy our work because we go and meet people’

A third key action was to ensure that VHWs were motivated; without this, multiple stakeholders, including VHWs themselves, stressed that the routinization of their various tasks and retaining them would be difficult. This was done through supervision, stipends and nonfinancial incentives. The scheme’s approach to supervision was adapted over time. Initially, VHWs received weekly supervision from CHEWs. However, interviewees representing the government suggested that CHEWs had been slow to fulfil their supervisory roles, leading to additional levels of supervision being introduced, based on existing actors and structures, specifically: LGA officers, who observed VHWs’ work and collected monitoring data during monthly house-to-house visits and Agency staff making quarterly supervision visits. Regular review meetings were introduced to provide VHWs with refresher training, and in each LGA, the Agency formed Management Committees to oversee the scheme, comprising LGA staff, community leaders and WDC members. Every quarter these committees reviewed monitoring data and addressed problems jointly with CHEWs and VHWs. Our government respondents from different levels of the health system and representing the nongovernmental implementer were supportive of this multi-layered approach since it ensured VHWs’ work met appropriate quality standards. VHWs said they valued opportunities to receive updated information and training and were motivated by multiple lines of supervision, which institutionalized their roles within the health system. Nevertheless, sustaining this approach was challenging in practice. For example, in remote areas, CHEW supervisors said that they faced problems with travelling long distances over difficult terrain, which VHWs themselves also experienced.

Additional incentives were also important. Stipends were particularly crucial, although VHWs and other respondents reported that early in the scheme, these were deemed too low and did not reflect their substantial workloads. An adaptation made in January 2018 was to increase stipends from 4000 Naira (11USD) monthly to 6000 Naira (16.5USD). Respondents representing the government and the nongovernmental implementer, as well as VHWs themselves, welcomed this; for example: ‘[It’s] a major boost to the project and a motivation to the VHWs’, said a nongovernmental officer. Subsequently, nonmonetary incentives were introduced, including 3 months’ maternity leave. Backpacks were also issued, making it easier to carry and protect equipment, and concerns about short-length hijabs being culturally inappropriate were addressed: ‘…the husbands frowned on the shorter length hijabs. So, we had [longer] hijabs made…’, a nongovernmental implementer noted. VHWs said that community acceptance motivated them, and they enjoyed this aspect of their work: ‘We enjoy our work because we go and meet people that we don’t know in some of these neighbouring villages’. Gaining new knowledge, and the gratitude of communities, were important incentives. A VHW summarized: ‘The programme has raised our status and we feel important’.

### Institutionalization within the health system: ‘what we enjoy most is being part of the health system’

A fourth key action promoting scheme’s sustainability was to institutionalize it within the health system. This took time; respondents from the government and the nongovernmental implementer indicated that institutionalization was needed at different levels of the system and multiple stakeholders were involved in the transition process. These respondents highlighted a number of mechanisms through which the scheme became institutionalized. One was to include the scheme as a line item in the state health budget. Another was to establish the management committees to oversee the scheme within each LGA. The involvement of LGA officers in supervising and monitoring VHWs also helped to formally connect VHWs to the wider health system: ‘[They] are the real approach to sustainability’, explained a government official. According to VHWs, efforts to link them to other health worker cadres and health system structures meant they were quickly accepted as fully institutionalized with the health system, rather than add-ons. One VHW said: ‘What we enjoy most is being part of the health system’. CHEWs reported that they had early concerns that VHWs might encroach on their jobs; however, close co-working changed this attitude: ‘They are our colleagues’, a CHEW participant affirmed. VHWs also noted that other cadres’ recognition of their effectiveness in their jobs helped to reinforce their acceptance. For example, primary facility staff began asking VHWs to transmit healthcare messages at immunization and antenatal clinics and sometimes to support women in the early stages of labour. One VHW confirmed: ‘It’s the importance of the work that has created a strong relationship between us and…[facility staff]’. Nevertheless, VHWs acknowledged that more awareness was needed they were to be fully accepted as legitimate, professional health workers, particularly within higher level facilities where some staff assumed VHWs were simply a new form of traditional birth attendant.

### Managing financial uncertainties: ‘a change in government can be the end of this beautiful dream’

A fifth action was to ensure that funding would continue. Government officials and nongovernmental implementers raised concerns about the scheme’s fragile financial sustainability after donor funding finished amid uncertainty about the outcome of the Nigerian general elections in February 2019. They highlighted that without continuity of political support, funding and the scheme’s continuation, would be in doubt: ‘…a change in government can be the end of this beautiful dream’, a nongovernmental implementer warned. These respondents suggested that the timing of the transition was not optimal; it would have been advantageous if the nongovernmental implementer’s grant had continued through the third-quarter of 2019, while the new government settled in. A government official commented: ‘The [externally funded] programme is exiting at the exactly the wrong time’. Our respondents pointed to a number of actions that helped to mitigate these financial uncertainties. The state’s existing 5-year strategic health plan that was due to end in 2022 provided some stability. Before that, the Agency could entreat political leaders to cover the scheme’s costs from the state’s budget by presenting its benefits and impacts to sympathetic state politicians: ‘What it would take to get there is, let the Governor see how this Scheme covers a huge gap [in] human resources’, explained a nongovernmental implementer. Additionally, there was considerable early deliberation about ensuring the scheme was framed as closely aligning with state and national maternal and child health reform priorities reflected in the state’s 5-year plan. Government officials and nongovernmental implementers agreed that maximizing the scheme’s institutionalization within the health system ahead of the elections also helped, since it would not be viewed as an external, donor programme. Indeed, considerable efforts had been made to ensure the scheme should not resemble: ‘…a donor-led, donor-owned programme’, as a nongovernmental implementer put it.

Notwithstanding these efforts, respondents suggested that limited financial resources at the state’s disposal would always threaten the scheme’s sustainability, including paying VHWs’ stipends, supervision costs and commodity supply: ‘…the major weakness is resources’ acknowledged a government interviewee. Despite increasing, stipends were not considered sufficient and did not cover all transport costs; many VHWs used their own money, resulting in some not visiting remote locations, with the danger they might falsely report activities: ‘..if you cannot give them money, then the data can be cooked…’, observed a nongovernmental implementer. Respondents doubted whether all of the supervision arrangements were sustainable, since, as additional levels of supervision were introduced, corresponding budget lines were not added. Similarly, uncertain funding for medicines and equipment required by VHWs meant that, at the time of our study, it was unknown whether beneficiaries would continue to receive free medicines when donor funding ended.

### Fostering community ownership and acceptance: ‘they are part and parcel of decision-making’

A final key action was fostering community ownership and acceptance, and respondents across the health system agreed that WDCs were a key platform for achieving this: ‘We have responsibility for taking care of the Scheme because it’s meant to help us’, a WDC member summarized. WDCs played multiple roles in the scheme. They monitored VHWs’ work and VHWs and CHEWs said they routinely consulted WDC members and worked together to solve local challenges. Committee members also contributed to decision-making about the scheme, and usually comprised of high-status members of their communities, which was important: ‘Most…are influential people from the community; they have a say’. For example, WDCs pushed to relax the VHW selection criteria and assisted in VHW selection. Some were active in advocating the government to finance the scheme in the longer term: ‘They are part and parcel of decision-making; we also plan with them’, a government official acknowledged. Their members also served as interlocutors between government and communities and hence maintained a flow of information about the scheme: ‘The WDCs are the window to the community!’ remarked a government official. Multiple respondent groups agreed that WDCs helped to promote community acceptance of VHWs and encouraged improved health-related behaviours and increased demand for healthcare. VHWs, WDC members and other respondents clarified that building community acceptance had taken time; early on some VHWs were not perceived as legitimate health workers: ‘You have to be diplomatic before you can convince them’, reflected a young VHW who struggled to persuade people that she had the knowledge to support pregnant women. Over time, by sensitizing community leaders, husbands and mothers-in-law, resistance diminished: ‘On our first day of visits, the community members mocked some of us…[but] we endured and…now we’re accepted and have become friendly with them’, a VHW reported.

VHWs and WDC members highlighted how communities started to accept VHWs’ competence and valued receiving health messages from literate women and liked the ‘modern way’ they worked: ‘The Scheme’s accepted beyond expectations’, said one WDC member, while a nongovernmental implementer remarked: ‘They’ve touched the lives of villagers and rural dwellers!’. VHWs said that this motivated them to continue performing their roles. Our respondents also noted the positive effects on communities’ health-related behaviour, and particularly in increasing healthcare uptake: ‘We…know they’re doing a good job, because we’ve seen the up-turn in [women attending] the facility’, explained a CHEW. While the scheme had positive effects, respondents from multiple stakeholder groups suggested that were also unintended consequences. Increased demand put pressure on parts of the health system; shortages of staff and medical supplies within primary healthcare facilities were described as insufficient to meet increased demand for antenatal care and facility deliveries. A nongovernmental implementer acknowledged: ‘There’s a point where if you raise demand and there’s no supply, then [the system] isn’t working…’.

## Discussion

Our study highlighted six key actions that were critical underpinnings of the VHW Scheme’s sustainability: strong government ownership with a planned transition of responsibilities; reflection points and adaptation; taking steps to motivate VHWs; institutionalizing the scheme within the state health system; anticipating and managing financial uncertainties; and embracing strong community ownership and acceptance.

This paper adds evidence to a body of literature seeking to explain the sustainability of donor-funded health interventions, including those that introduce and strengthen community health workers. A country-driven approach was critical for the scheme’s sustainability: stakeholders went to great lengths to avoid the scheme being introduced in a top-down, donor prescribed fashion, which is a common problem with donor-funded interventions that limits their sustainability (Spicer *et al.*, 2018; Wickremasinghe *et al.*, 2018). Several other studies of sustainability also highlight the importance of buy in by government and other stakeholders, including Braithwaite *et al.* (2018); Chambers *et al.* (2013); Hirschhorn *et al.* (2013); Iwelunmor *et al.* (2016); Larson *et al.* (2014); Sgaier *et al.* (2013); Shediac-Rizkallah and Bone (1998); Shelton *et al.* (2018); Stirman *et al.* (2012); Torpay *et al.* (2010). Our study highlights how critical this was: the scheme was presented as government-led, with support from a nongovernmental implementer with donor funding, and a genuine partnership existed between the state government, the nongovernmental implementer and the donor involving joint decision-making at each stage of planning and implementation, and government’s responsibilities were clarified in advance as part of the phased transition process (issues also raised by Sgaier *et al.*, 2013; Pallas *et al.*, 2013; Wickremasinghe *et al.*, 2018). Nevertheless, strong government ownership can sometimes be challenging to achieve. For example, financial uncertainties linked to control of funds lying with the Ministry of Finance created problems for the VHW Scheme. Hence, our study illustrates the need for implementers to develop strong relationships across government departments both within and beyond the health sector (Schell *et al.*, 2013). Indeed, this problem is not unique to this scheme; the introduction of community health worker interventions in other low-resource settings has faced similar problems (for example, Black *et al.*, 2017; Strodel and Perry, 2019).

In addition to government ownership, community ownership has been raised as contributing to the sustainability of health interventions supported by donors, for example, by Iwelunmor *et al.* (2016) and Strodel and Perry (2019). In Gombe community ownership was formally institutionalized within the health system through the WDC structures, which reinforced the scheme’s links with the health system and involved communities in decision-making about the scheme. The WDCs were also well-placed to hold government and VHWs to account, which further contributed to country ownership, and hence to the scheme’s sustainability. A number of writers also reflect on the importance of dynamic rather than static approaches to designing interventions for sustainability (for example, Chambers *et al.*, 2013; Scheirer and Dearing, 2011; Shediac-Rizkallah and Bone, 1998; Shelton *et al.*, 2018). Our study resonates with those studies, and in addition highlights the value of hard-wiring flexibility and adaptation into the joint planning and implementation of an intervention. For the VHW Scheme, this was done by government and other stakeholders agreeing to introduce pause points to draw out practice lessons to enable collective reflection and to jointly agree adaptions to the intervention’s design to ensure it closely responded to the contexts into which it is introduced: what stakeholders of the VHW Scheme called the ‘adaptive management process’.

Hence, we argue that donors introducing interventions should do so in response to government requests for support, with broad stakeholder ownership and buy-in, and closely reflecting local contexts rather than be rigidly prescribed. Health interventions therefore need to be seen as country programmes supported by donors, rather than donor programmes introduced into countries.

### Limitations

The study has some limitations. Data collection was limited to two wards and two LGAs, constraining our scope for making generalizations, although interviewing LGA and state-level stakeholders mitigated this because they could reflect on other wards and LGAs. Additionally, it was not possible to study the scheme beyond December 2018 and thereby document the situation when the Agency took over full responsibility. The researchers involved in the study acted as the scheme’s ‘critical friends’, which enhanced access to research participants and meant understanding and trust could be built (Coghlan and Brydon-Miller, 2014). The research team consisted of members of the first author’s institution and an independent research consultancy based in Abuja, Nigeria. While the evaluation was commissioned by the intervention’s funder, the Bill & Melinda Gates Foundation, and some members of the research team were already known to some of the research participants, the research team was given complete freedom by both the funder and by the scheme’s stakeholders to design the study, define study questions and parameters, to analyse and interpret data and to report findings. Indeed, the funder did not play any role in conceptualizing and designing the study, and in analysis, interpretation and reporting of our data. Nevertheless, the authors’ perspectives and experiences inevitably shaped the study and our interpretation and reporting of the data. While the fact that some members of the research team were known to some research participants may have limited our ability to assess more critical and negative aspects of the scheme, we have endeavoured to present both positive and negative reflections on the scheme. Indeed, the main aim of this paper is to highlight the factors promoting sustainability, rather than describe challenges or failures.

## Conclusions

For a health intervention to be sustainable, it is important to plan for reflection and adaption points from the outset, and government and community ownership and a phased transition of responsibilities are crucial. Our study adds to a body of evidence highlighting the difficulties stemming from donors working in top-down ways; donors need to avoid unilaterally introducing health interventions, and instead should respond to country priorities and government requests for support, and work in genuine partnership with country governments. Hence, health interventions need to be seen as country programmes supported by donors, rather than donor programmes introduced into countries.

## Data Availability

The data that support the findings of this study are available on request from the corresponding author, [NS]. The data are not publicly available due to them containing information that could compromise the privacy of research participants.

## Abbreviations

The Agency Gombe State Primary Health Care Development Agency

CHEW Community Health Extension Worker

CHIPS Community Health Influencers Promoters and Services

LGA Local Government Area

VHW Village Health Worker

WDC Ward Development Committee
